# Response to comments on the tolerance to Clostridioides difficile spores to sodium hypochlorite disinfection

**DOI:** 10.1099/mic.0.001463

**Published:** 2024-05-21

**Authors:** Humaira Ahmed, Lovleen Tina Joshi

**Affiliations:** 1 Peninsula Medical School, Faculty of Health, University of Plymouth, Devon, PL4 8AA, UK; 2 Peninsula Dental School, Faculty of Health, University of Plymouth, Devon, PL4 8AA, UK

**Keywords:** *Clostridioides difficile*, chlorine, disinfection, evolution, resistance

**Dear Editor,**

We are pleased that the article we wrote on the *in vitro* effectiveness of sodium hypochlorite on *C. difficile* spores has raised important debate [1]. We welcome much needed academic discourse on the topic of biocide tolerance, which we feel, has been overlooked as a research area relevant to reducing spread of infections and antimicrobial resistance [2,3].

We write this letter not only to respond to comments made; but to also highlight the importance of basic scientific research evidence, as opposed to only clinical research, in understanding the behaviour and characteristics of microorganisms. Clinical research should be used in combination with *in vitro* fundamental biological research to answer fundamental questions about the biology of infectious microorganisms. Clinicians and laboratory scientists should be working together to tackle healthcare challenges. The research we conducted adds to a body of knowledge that others can build on and comments made about our research have been addressed below.

We undertook this study in response to the United Kingdom Department of Health guidelines on disinfection of *C. difficile* spores [4] and sought to examine whether these guidelines were fit for purpose given that microorganisms evolve, and that *C. difficile* spores are difficult to eradicate [5]. We seek to answer questions about *C. difficile* spore structure, adherence, and morphology in response to biocide exposure.

We would like to highlight that the majority of comments made by Cadnum *et al*. [6] and Decousser *et al*. [7] have already been addressed within the discussion section of our paper; we undertook careful critical analysis of the research findings and did not overstate the impact of the research in a clinical setting (which is what both commentaries appear to be alluding to) [6,7]. We also pointed out in our publication that we are already looking at the effects of pH, temperature and are undertaking time-kill assays as part of our ongoing unpublished research. As academic researchers, we have clearly acknowledged the limitations of our study and the fact that more research into disinfection tolerance of *C. difficile*, and the molecular mechanism of biocide damage, needs to be conducted.

At present there is large gap in the literature with regards to the response of *C. difficile* spores to biocide disinfection; past studies have used a *Bacillus subtilis* spore model to understand whether and how bacterial spores are killed by biocides [8,11]. Of course, Bacillus species and Clostridial species are biologically different; for example, *Clostridioides difficile* is an obligate anaerobe, causing potentially fatal antibiotic-associated diarrhoea and is largely transmitted in clinical environments, and Bacillus species are aerobic and regulate sporulation, germination, and spore coat development differently to *C. difficile* [12,15]. The biocide response of *C. difficile* spores has not been well characterised to date.

However, our observations into the biology of *C. difficile* spore adherence, biocide tolerance and persistence on hard and soft surfaces is not singular; other academic researchers have also observed this phenomenon [16,17]. Moreover, the statement that *‘The inoculum of 10^8^ spores used for the transfer to surgical scrubs and to patient gowns corresponded to a significant amount of spores that is not supported by any clinical claims*’ in lines 53–54 of Decousser *et al*., [7] is perhaps true clinically; however, that does not mean we cannot undertake basic scientific research to plug literature gaps and model this in a laboratory research scenario. Therefore, the point of this line of query remains unclear.

In response to Decousser *et al.*’s point that our research is irrelevant because of apparent washing of personal protective equipment (PPE) and soft surfaces at 60 °C: ‘*Furthermore, because of the lack of knowledge about the sodium hypochlorite putative action in this condition combining different factors (i.e. the penetration of hypochlorite in the personal protective equipment (PPE) and/or its interactions within the material), and given that such reusable PPE are expected to be laundered (with 60°C-washing, drying and high temperature ironing), the transposition of this experiment to the real-life hospital conditions appeared irrelevant’* frankly demonstrates an acute lack of knowledge about the widely documented ability of *C. difficile* spores to survive the hospital laundering process [18,20]. The point made is refutable; there are many studies that have shown *C. difficile* spores survive the laundering process and we would encourage any interested parties to please read those peer-reviewed published papers before making assumptions about the efficacy of laundering for *C. difficile* disinfection.

Comments have been made on social media platforms in the USA regarding the relevance of this research given that EPA- registered (Environmental Protection Agency) chlorine releasing disinfectant was not used in the study. We did not undertake the study to verify a product; we undertook the study to fundamentally *understand* observable phenomena where spores of * C. difficile* are tolerating chlorine releasing agents based on UK guidelines [4].

*C. difficile* disinfection guidelines in the USA are regulated by the EPA, while globally there are other regulatory bodies who provide varying guidelines for *C. difficile* disinfection. This means that research may not always use EPA-approved biocides or formulations, and therefore it is important for research to accommodate varying scientific methodology [21]. By being inclusive in our research we can avoid inadvertent gatekeeping of important scientific information.

Questions have been asked about why we diluted sodium hypochlorite with sterile deionised water and have stated that this is not the method used in the ‘real world’. In return, we firstly ask whether cleaners (environmental service workers) in hospitals globally are trained to use disinfectants properly, and whether the chlorine releasing product is used fresh (and freshly diluted) when cleaning takes place, or if the correct biocides are being used generally [22]? Has anyone monitored this in practice? To our knowledge these types of study have not widely taken place, and we would urgently recommend that appropriate training is given to cleaners to ensure cleaning methodology, and use of biocides, is appropriate. There is also a question about whether hospitals have the funds to employ effective terminal disinfection measures or whether cost cutting has been implemented [23]. The point is that because we cannot definitively answer any of the above, we cannot assume that disinfection in clinical practice is perfect; hence why our study is important.

Secondly, we have also noticed that many studies in the area of biocides do not tend to neutralise the biocide activity after a set period of time [24]. This is important to model because in practice, biocides such as chlorine releasing agents lose stability quickly. Also, the majority of laboratory-based studies have used sterile deionised water or phosphate buffer saline (PBS) as a diluent when testing chlorine releasing agents against spores [9,16, 17, 25, 26].

Moreover, there are no guidelines (EPA or other) associated with disinfection of soft surfaces (such as hospital beds, mattresses, bedding and PPE). This is a major regulatory oversight given the numerous studies conducted into hard and soft surface vectors of *C. difficile* transmission in hospitals [27,29].

Our *in vitro* studies into *C. difficile* spore biology and tolerance to chlorine releasing agents on hard and soft clinical surfaces have been ongoing since 2012 [30,33]. Recently, we also undertook a collaborative biophysical study [33] to understand how certain *C. difficile* spore ribotypes can tolerate disinfection with sodium hypochlorite using Raman spectroscopy methods. Our latest paper [1] builds upon this body of research and has sought to understand how and why we have observed chlorine biocide tolerance in *C. difficile* spores.

The point made by Decousser *et al*., states that our 2023 [1] paper conflicts with results in the biophysics paper [33]; however, we did not use the exact same methodology. There is a crucial difference in spore production with the density gradient material we used; sucrose in Malyshev *et al.* [33] and Histodenz in Ahmed & Joshi (2023) [1,32]. This may have resulted in variations in spore exosporium morphology and adherence ability, which may account for the variations we saw. Moreover, we used a very high concentration of spores in these experiments (1×10^9^ c.f.u. ml^−1^) and did not seek to disperse the spores in solution using sonication for example, we used vortex mixing. This may have resulted in spore aggregation when exposed to the biocide, reducing the spore surface area exposed to biocide. This is another critical point as one of our working hypotheses is that the spores are aggregating in solution, where the ‘sticky’ exosporium causes individual spores to stick and clump together, forming a type of ‘biofilm’ which can physically resist biocide attack. This is a phenomenon we intend to explore in further studies and could explain what is happening in solution at the spore and exosporium level.

It is true that we do not understand how sodium hypochlorite (NaOCl), or sodium dichloroisocyanurate for that matter, target the spore and cause it to be inactivated. This is important research that must be conducted. What we do know is that varying genes within *C. difficile* spore DNA do encode chemical, heat and biocide tolerance. These include: *cotA*, an exosporium gene encoding lysozyme, ethanol and heat resistance; *sodA* a spore coat gene with roles in biocide resistance to Virkon, ethanol and hydrogen peroxide, and the small-acid soluble protein *sspB* genes which have been linked to UV resistance [1,34]. Therefore, we are pleased that Cadnum *et al*. [6] acknowledged this point in their comment stating *‘Finally, given the high genomic fluidity of ribotype 027 C. difficile strains, we cannot exclude the possibility that the R20291 isolate tested by Ahmed and Joshi has accumulated genetic changes resulting in reduced susceptibility to chlorine-releasing disinfectants’*, which is a point we had made within our paper [1,6]. However, we also wish to point out that if we are seeing genomic evolution in the laboratory, it may be likely this is occurring in clinical setting; meaning studies into the efficacy of terminal disinfection on *C. difficile* contaminated wards, using Whole Genome Sequencing (WGS), should be conducted over a sufficient period of time to determine if this is happening in clinical settings. This all adds to the body of knowledge in the area and can help to tackle the issue.

The experiments undertaken were not conducted to match EN17126 procedures because we were asking academic questions to understand spore biology; the EN17126 test evaluates sporicidal efficacy of a product against a single strain of *C. difficile* spores (according to European guidelines the NCTC 13366 strain), *Bacillus subtilis* and *Bacillus cereus* [35]. Comments made by Decousser *et al*., state that (a) *‘First, the choice of the strains was restricted to only two different PCR-ribotypes (027 and 012); the results of the experiments cannot be generalizable to the highly diverse population of CD*’ and (b) *‘In addition, they did not use a normalized procedure (e.g. the EN 17126) to prepare the spores suspension and to measure the sporicidal activit*y’; however, it is reductive to critique our use of two ribotypes of *C. difficile* in this study when the EN17126 test only uses one. Surely that *feeds into* the argument that the EN17126 test is not currently fit for purpose and may need to be adjusted to account to test for more representative strains of *C. difficile* spores; especially when varying strains across the five evolutionary clades produce diverse spore populations (e.g. genetically or phenotypically in terms of exosporium presence and morphology) [5,36]. We did study two strains of Ribotype 027 and 012 because these strains are hypervirulent and genome sequenced, making genetic analysis easier. Therefore, as hypervirulent strains cause more severe infections it can be argued that this research is more clinically relevant currently than the standard EN17126 tests. Therefore, when considering the above context, the argument is presented by Decousser *et al*., appears weak.

More research needs to be undertaken in an academic and clinical context to truly understand the implications of *C. difficile* tolerance to chlorine releasing agents. However, we do acknowledge that more studies need to be conducted across a range of strains (clinical and laboratory based) to gain a better understanding of tolerance levels. For this to happen however, the research and Infection Prevention and Control (IPC) community must take the issue of biocide resistance seriously and accept that there is space for fundamental scientific academic research examining spore biology.

Any research produced in the area of disinfection should be impartial and avoid personal or company interests. One has to question the influence of industry in potential censorship of academic research, or their ability to discredit academics that conduct research which is not in their commercial interests. Such influences can stunt academic freedom of speech and freedom to conduct research into these ‘controversial areas’ causing conflicts of interest to arise [21,37]. It is an inconvenient truth for some (e.g. in hospitals and the IPC industry) that laboratory research has found chlorine tolerant *C. difficile*; however, rather than actively suppress and discredit this type of research, we should examine the future implications. Indeed, it is easy to argue that industry influence has stunted progress within core areas of the science; a prime example being the broken economic market for antimicrobial development [38].

The impartiality and neutrality of Cadnum *et al*., can be questioned as they have openly acknowledged [6] being funded by EcoLab, Clorox and Pfizer (some of whom produce chlorine products). The Joshi laboratory group have not been funded by any such parties to date and remain impartial. We do not dispute that some company formulations that contain chlorine releasing agents may be effective, as shown in Cadnum *et al*. [6], but that does not mean that *C. difficile* evolution and laboratory study should be ignored.

It is clear, however, that chlorine tolerance raises important questions about using the right biocide for the specific microorganism, at the right time and at the right concentration for effective disinfection. This is a key pillar of IPC. Ineffective use of biocides can cause selective pressure which increases tolerance of bacteria to biocides and antibiotics; these stress responses can trigger evolutionary changes within the genome of the microorganism [2,3]. This critical information also appears to be overlooked. Alternative disinfection solutions for *C. difficile* should be explored rather than ignore the issue. Effective solutions include ultraviolet light (germicidal and far UVC), hydrogen peroxide vapour, hypochlorous acids, antimicrobial surfaces and using a combination of disinfection methods [1,3].

To truly progress and tackle global challenges, like infection spread and antimicrobial resistance, we must continue to question current practices and procedures and ensure they remain fit for purpose. Let us bridge the gaps between disciplines and keep informed of current research in the area. It sounds like a cliché, but academics, clinicians, hospital trusts and industry must work together for the benefit of all.

## Abbreviations

EPA: environmental protection agency
PPE: personal protective equipment
